# Endovascular repair of aortic pathologies involving the aortic arch using castor stent-graft combined with in-vitro fenestration technology

**DOI:** 10.1186/s12872-023-03138-6

**Published:** 2023-02-24

**Authors:** Zeng-Rong Luo, Jia-Xin Zhang, Zhong-Yao Huang, Liang-Wan Chen

**Affiliations:** 1 Department of Cardiovascular Surgery, Fujian Medical University Union Hospital, Fuzhou, 350001 People’s Republic of China; 2 Key Laboratory of Cardio-Thoracic Surgery (Fujian Medical University), Fujian Province University, Fuzhou, Fujian Province People’s Republic of China

**Keywords:** Supra-aortic branches, TEVAR, Reconstruction, Castor, In-vitro fenestration

## Abstract

**Background:**

Aortic arch pathologies are concerning clinical conditions with poor prognoses. The use of thoracic endovascular aortic repair (TEVAR) has been investigated to treat aortic arch pathologies. Nonetheless, cerebral blood flow regulation during endovascular aortic arch repair therapy remains challenging. Castor, a unique single-branched stent graft, has been proven effective for retaining the left subclavian artery (LSA). This study aimed to determine whether endovascular therapy for pathologies involving the aortic arch using Castor in combination with the in-vitro fenestration technique is promising, effective, and safe.

**Methods:**

Eligible patients were enrolled between June 2018 and December 2021. All patients underwent TEVAR with an evaluated proximal landing zone for “Castor” located in Ishimaru zones 0–1. Moreover, the supra-aortic branches (SABs) were reconstructed using the Castor in combination with the in-vitro fenestration technique.

**Results:**

Herein, 57 patients with aortic arch lesions were treated with Castor in combination with the in-vitro fenestration technique. Innominate artery and the left carotid artery (LCA) were reconstructed in 5 patients, LCA and left subclavian artery (LSA) were reconstructed in 22 patients, and the total SABs were effectively reconstructed in 30 patients (including a hybrid arch repair case). Among them (excluding a hybrid arch repair case) were in-vitro fenestration methodologies for LCA in 32 of 34 cases (2 switched to in-situ fenestration) and LSA in 51 of 56 cases (3 switched to in-situ fenestration and 2 converted to spring coil caulking); furthermore, LCA and LSA in-vitro fenestration were simultaneously successfully performed in 27 of 34 cases. There were no surgical-related neurological complications, and early mortality was estimated at 5.26%. At a mean follow-up of 3.75 months, computed tomography (CTA) images confirmed that each branch stent remained patent. There were no signs of endoleaks, migrative manifestations, or the need for secondary endovascular intervention or conversion to open surgical procedures.

**Conclusion:**

Castor, in combination with in-vitro fenestration, reflects a feasible, efficient procedure for re-developing SABs.

**Supplementary Information:**

The online version contains supplementary material available at 10.1186/s12872-023-03138-6.

## Introduction

Aortic arch pathologies include dissection, aneurysm, intramural hematoma, and aortic ulcer involving supra-aortic branches (SABs). Aortic dissection and aortic aneurysms involving the aortic arch are particularly complex and catastrophic and are correlated with considerably elevated mortality and morbidity [1].

Aortic arch pathologies traditionally require surgical treatment to prevent organ mal-perfusion, swift aortic development, and aortic rupture. Nevertheless, surgical mortality and morbidity remain high for aortic arch pathologies, especially among elderly patients with substantial comorbidities, despite recent advances in surgical therapeutic techniques [2, 3]. The mortality and stroke rates during emergency surgery involving aortic arch were recorded as high as 15% [4].

Thoracic endovascular aortic repair (TEVAR) has been a fast-expanding technique during the last few years. Endovascular surgery can potentially reduce cerebral disorders, early mortality, and duration of hospitalization in comparison to open surgery due to its lower risk of invasiveness, less reliance on mechanical-based circulatory assistance, and lack of aortic cross-clamping [5, 6]. In this view, endovascular therapy can be an effective therapeutic option for minimizing surgical injury in high-risk patients who are poor or elderly and have a low tolerance for cardiopulmonary bypass/hypothermia, provided that a comprehensive pre-surgical evaluation confirms the absence of coronary artery disease/aortic regurgitation [7].

TEVAR was initially used to treat thoracic aortic aneurysms (TAA), but it has since been developed to treat several types of aortic lesions. Presently, it is being evaluated as an endovascular treatment for arch lesions and even the ascending aorta [8–10]. Currently, with an emphasis on endovascular treatment of aortic arch lesions, the Ishimaru classification is utilized for dividing the aorta based on the landing zone of the proximal and distal attachments [11].

**Fig. 1:**
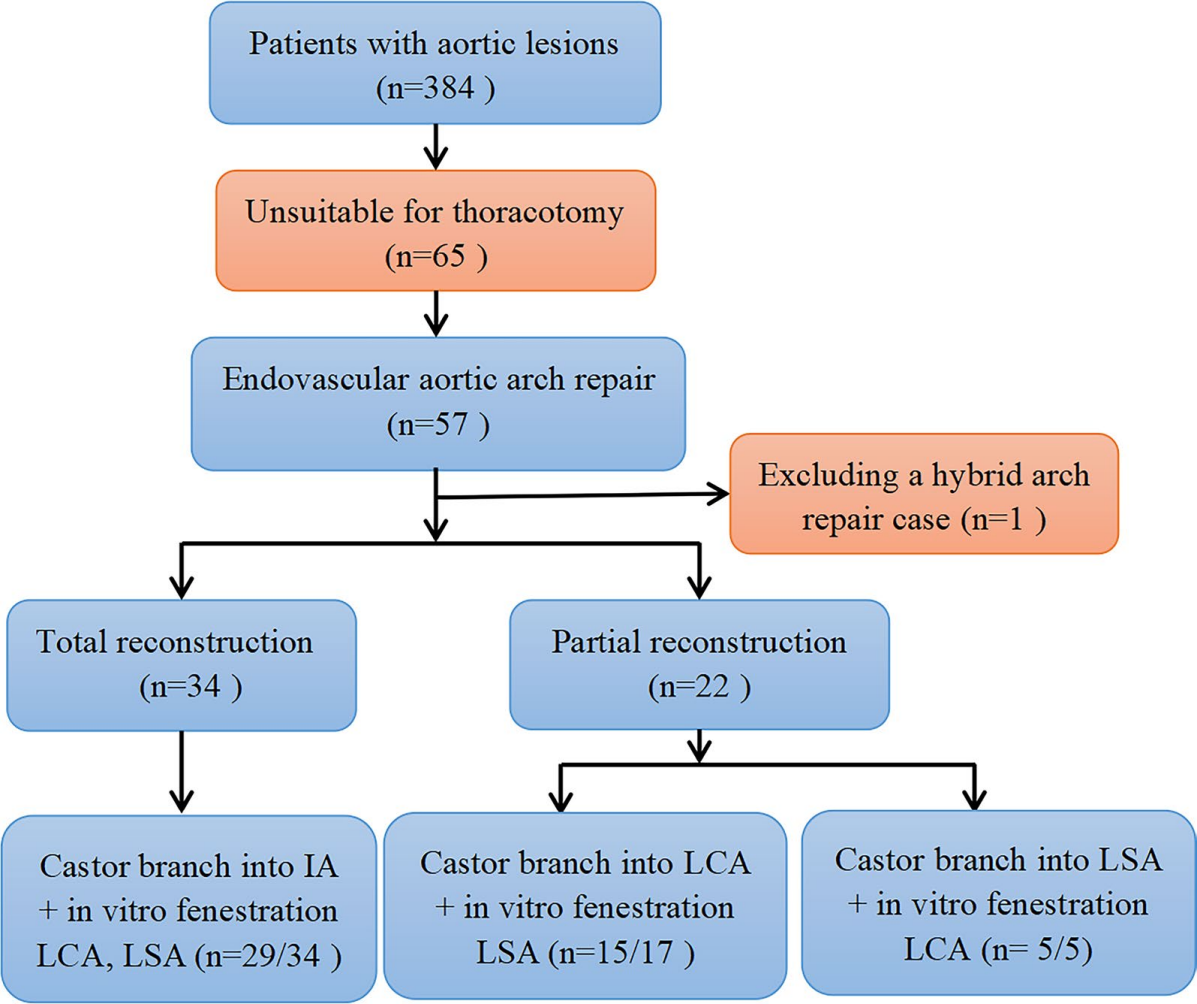
Flow chart of the study

In this study, the medical outcomes of patients with aortic arch pathologies who underwent endovascular repair using Castor, a unibody single-branched stent grafting, in conjunction with in-vitro fenestration between June 2018 and December 2021 were retrospectively analyzed. Additionally, data from patient follow-up was recorded. The purpose of our study was to evaluate the efficacy, safety, and efficacy of endovascular repair of thoracic-aortic lesions affecting the aortic arch using the Castor approach in conjunction with the in-vitro fenestration technique, as well as to reveal our initial experience with such a procedure (Fig. 1).

## Materials/methods

### Clinical cases/methodology

This investigation consisted of a sole-center retrospection-based evaluation comprising 57 patients who had endovascular treatment with Castor stent graft in combination with in-vitro fenestration at our institute from June 2018 to December 2021. A multidisciplinary panel assessed each patient recruited for TEVAR and decided they were unfit for open-heart surgery. A patient could be selected for endovascular surgery for multiple reasons, such as poor physical ability, advanced age, a severe health condition, or a history of cardiac surgery. The inclusion criteria were Stanford-type non-A non-B dissection, aortic ulcer, intramural hematoma, and aortic arch aneurysm (AAA) affecting orifices of SABs. In other words, the proximal landing zone must locate at the Ishimaru zones 0–1. The following patients were not included in the study: (1) cases having severe peripheral vascular disease/small-diameter femoral artery access; (2) those in which maximum stent size could not completely close the lesions; (3) those with severe coronary artery or aortic valve disease; (4) aortic arch and SABs anatomical variations; and (5) bovine arch cases with short neck before branching BCA and LCA.

The digital subtraction angiography (DSA) imaging and computed tomographic angiography (CTA) data for each patient were obtained from our hospital's picture-achieving system. The electronic medical record system retrieved documentation, operation notes, and postoperative outcome information.

The surgical methods, difficulties that arose after surgery, and the final clinical outcomes were all meticulously recorded. This study focused on the risks associated with endovascular procedures, including stroke, endoleak, graft migration, paraplegia, retrograde aortic dissection, and mortality. The ethics committee of Union Hospital of Fujian Medical University waived the need for informed consent and approved this study (2022KY101, date: 2022-01-10). In addition, because this investigation was conducted retrospectively, participants were not required to provide informed consent.

### Surgical procedures

Cardiovascular surgeons with extensive experience and developed TEVAR skills performed endovascular surgical procedures at our institution. Castor, a novel unibody single-branched stent graft, was used in conjunction with in-vitro fenestration to perform the endovascular repair for aortic arch/SABs in this study.

### Castor

**Fig. 2:**
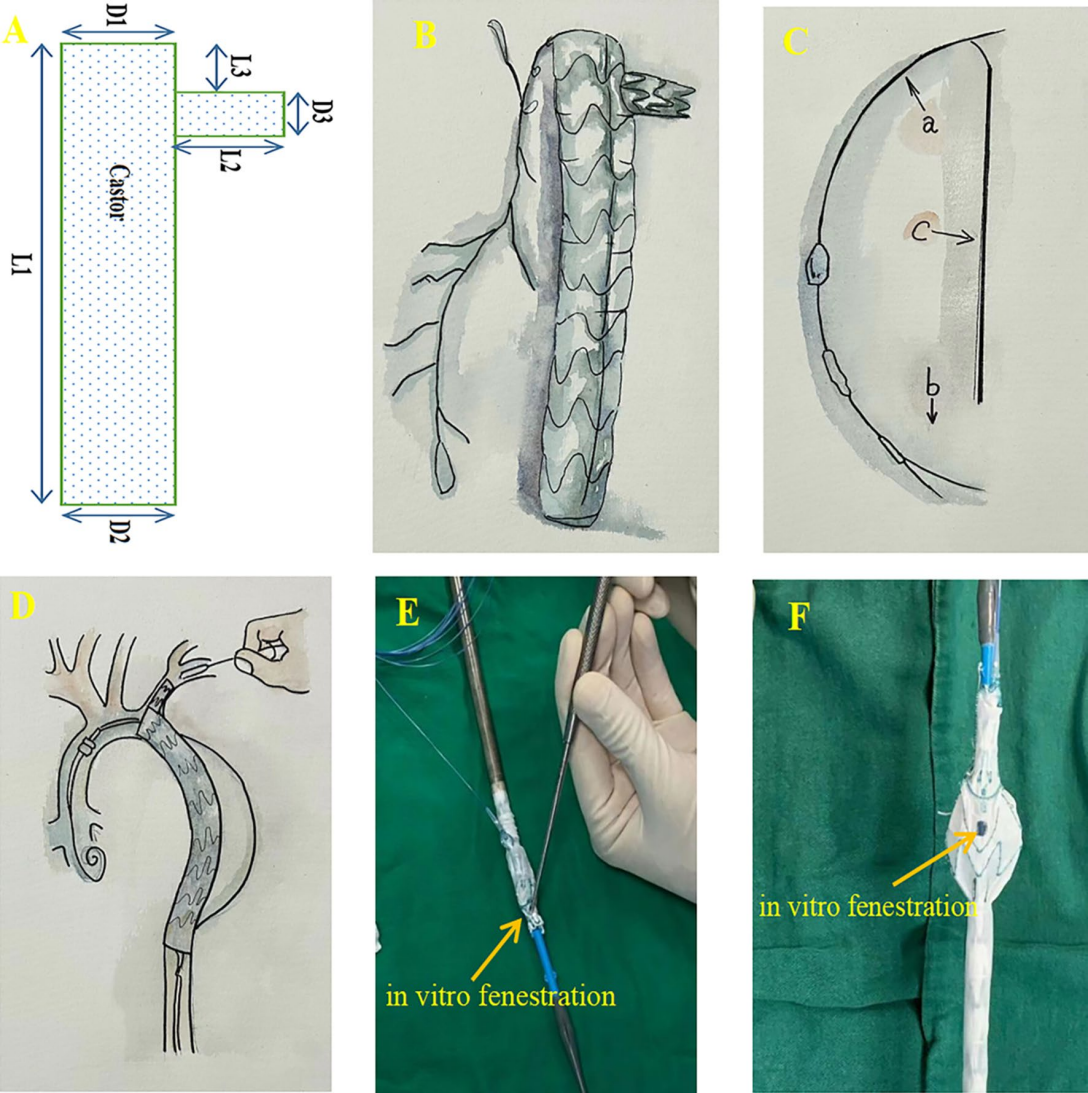
**A** The import size parameters of the Castor single-branched stent graft. Nine radio-opaque marks (small blue circles) are designed around the aortic trunk and branch section. **B** The structure of the Castor. Three shortwave amplitude brackets are designed in the proximal to increase the sealing ability. **C** The structure of the delivering system: (a) The outer sheath with low friction and hydrophilic coat; (b) nickel titanium trigger wire of the main graft trunk; (c) traction wire of the single-branch. **D** A steel ring is designed in the origin of the single-branch to trigger it open. **E** and **F** In-vitro fenestration was performed on the Castor main graft trunk

Castor single-branched stent graft (MicroPort Medical, Shanghai, China) was designed with a branch section to retain the LSA while sealing entry tearings (Fig. 2A). The Castor stent-graft system is built from woven polyester fabric bonded onto self-expanding Nitinol stents, lacking distal/proximal bare stents (Fig. 2B). Such a distribution platform comprised a 22 F exterior sheath (Fig. 2C-a) treated with a low-friction hydrophilic coat and inside soft polyester, fabric sheath encasing individually folded aortic transplant trunk/branch portion. The aortic transplant trunk is folded using thread loops/nickel-titanium wire (trigger wire; Fig. 2C-b). A "cap" made of polyester fabric coupled to a traction wire folds the branch portion, allowing the aortic trunk and branch section to be released independently (Fig. 2C-c). To maintain the branch segment's accessibility, a steel ring is connected to its origin (Fig. 2D). Subsequently, the Castor stent-graft's size parameters were established, and in-vitro fenestration was carried out following the intra-operative measurement data obtained during aortography (Fig. 2E, F).

### In-vitro fenestration

In-vitro fenestration technology can be developed from single fenestration to bilateral fenestration. Preoperative measurement and fenestration design are important. Accurate measurements should be made on the workstation before the operation to locate the fenestration position. The measured data include aortic diameter, arch angle, branch diameter, branch spacing, and angle. According to the measured data, an in-vitro fenestration is designed. In the meantime, the relative position of the stent ring steel wire and the in-vitro fenestration shall be determined based on the in-vitro fenestration design scheme, attempting to prevent the in-vitro fenestration from crossing the stent ring steel wire. After the in-vitro fenestration design is complete, reinsert the Castor main stent graft into the conveying system and attempt to avoid distortion and shortening. Moreover, the conveying system must have adequate control over the stent. In releasing the main Castor stent, the anteroposterior position mark is the most important. The steel wire segment of the stent ring can be used as a mark.

### Procedure

This study provides a basic description of the established method used in our center. The participant was positioned in a supine posture throughout the generalized anesthetic state, with the brachial, inguinal region, left upper extremity, and bilateral neck cleaned. Following this, incisions were made to expose the left brachial artery, common carotid arteries, and unilateral femoral artery. A shorter sheath (8-Fr) was used to access the left brachial artery, after which a pigtail catheter (5-Fr) was implanted into the aortic root for angiography. Angiography was performed to reconfirm tear position, lesion-implication level, dominant vertebral artery, and aortic arch diameter.

In our operation, we take different landing zones depending on the scope of the SABs requiring reconstruction.

**Fig. 3:**
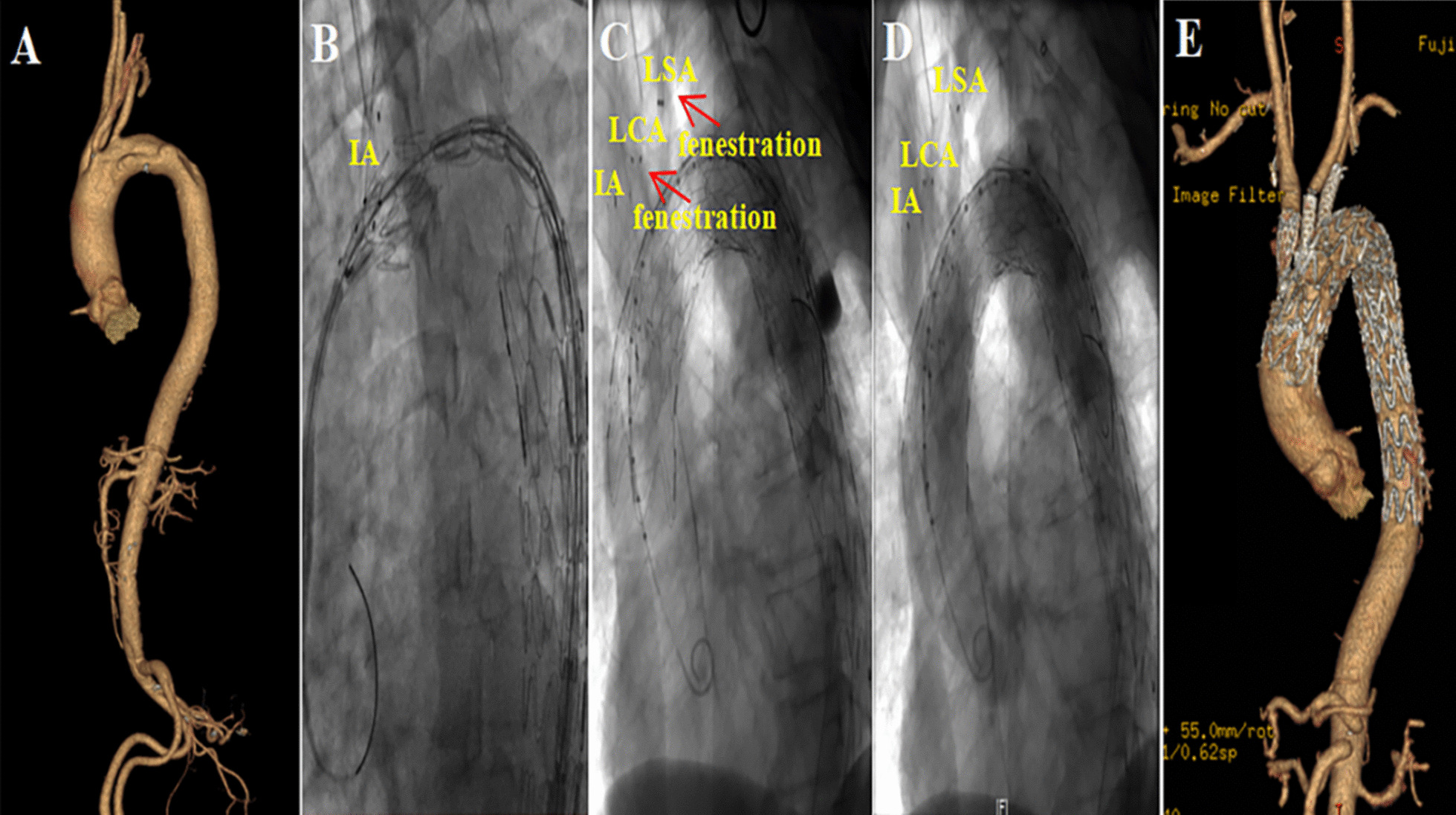
Total reconstruction of supra-aortic branches. **A** CTA display aortic pathologies involving the aortic arch. **B** The single-branch of “Castor” was pulled into the IA along the right IA track. **C** A covered short self-expanding stent graft of appropriate size was implanted and deployed in the in-vitro fenestration of LCA and LSA, respectively. **D** DSA show the perfect patency of supra-aortic branches. **E** Postoperative CTA show the perfect patency of the supra-aortic branches

**Fig. 4:**
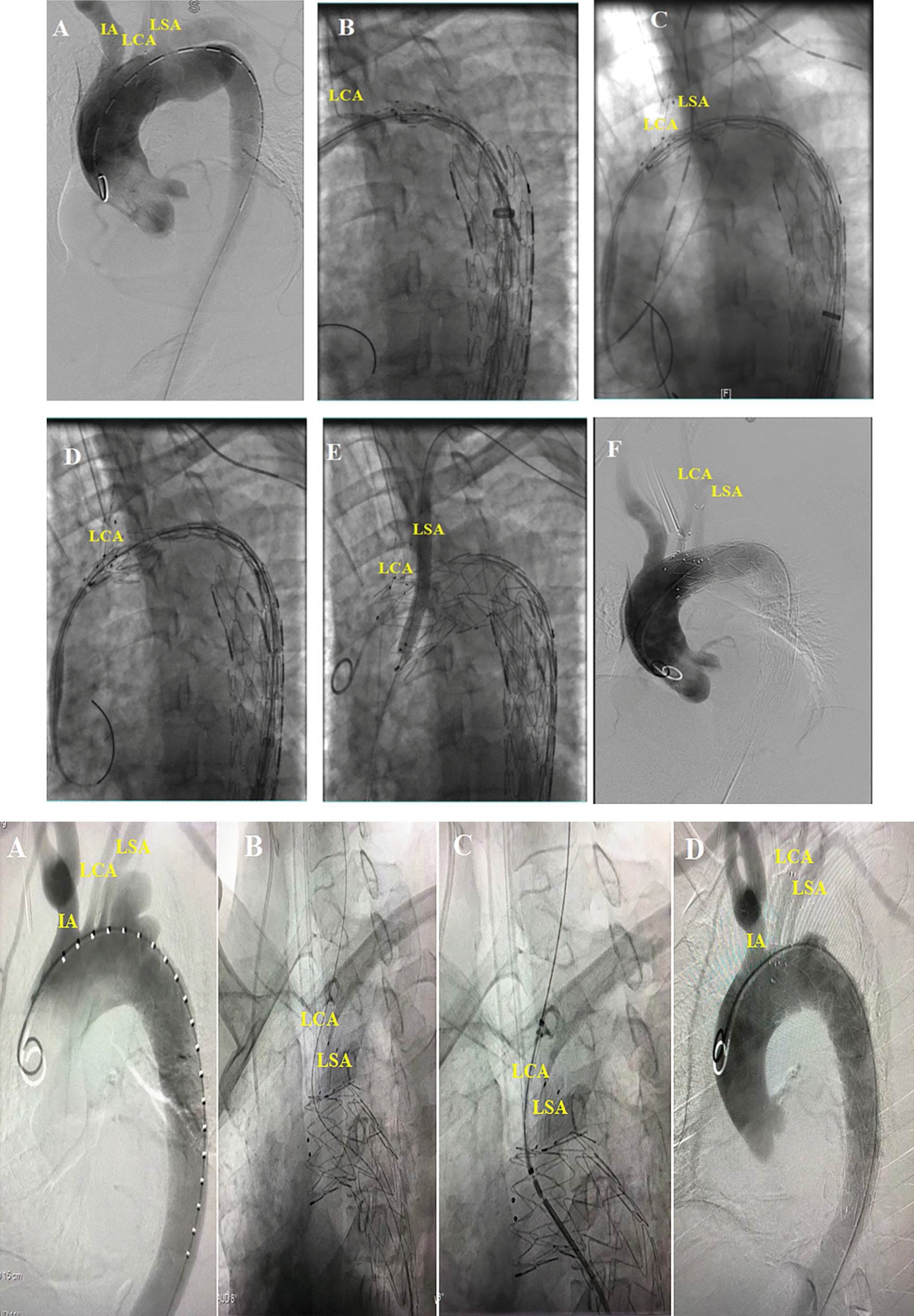
Partial reconstruction of supra-aortic branches (refers to the reconstruction of LCA and LSA). **4I** (LSA in-vitro fenestration): **A** DSA display aortic pathologies involving the aortic arch. **B**, **C** The single-branch of “Castor” was pulled into the LCA along the LCA track when sending the main body of “Castor” along the aorta track to the marked landing zone. **D** Release the main body and single-branch of “Castor”. **E** A covered short self-expanding stent graft of appropriate size was implanted and deployed in the expanded in-vitro fenestration of LSA. **F** DSA show the perfect patency of the supra-aortic branches. **4II** (LCA in-vitro fenestration): **A** DSA display aortic pathologies involving the aortic arch. **B** The single-branch of “Castor” was pulled into the LSA along the LSA track and was released, meanwhile a hydrophilic Stiff guidewire was switched in the LCA track through the in-vitro fenestration. **C** An expansion balloon was partly advanced through the in-vitro fenestration into the Castor main stent along the hydrophilic Stiff guidewire and deployed. **D** DSA show the perfect patency of the supra-aortic branches

1. *Total reconstruction*. When total SABs reconstruction is needed, the landing zone is located in Zone Z0 (Fig. 3). The sheath was inserted by retrograde puncture of the right common carotid artery and left common carotid artery, respectively. After the landing zone was clearly defined and marked by angiography, a good guide wire track was established along the aorta, right IA (in rare cases, the sheath was inserted by retrograde puncture of the right brachial artery, and guide wire track is established along the right IA via the right subclavian artery), and LCA. First, the main body of “Castor” was sent along the aorta track to the marked landing zone, where it was released instantly to cover the aortic arch lesion from the landing zone to the distal descending aorta. In the meantime, the single branch of “Castor” was pulled into the IA and released along the right IA track in an instant to restore IA perfusion immediately (monitoring the right upper limb's blood pressure and intraoperative cerebral oxygenation with the Regional Oximetry System [VISTA, Covidien]) and ensure the safety of the next step. Next, a Stiff hydrophilic guidewire was switched in the LCA track with an expansion balloon catheter that was partially advanced through the fabric covering into the Castor main stent via the in-vitro fenestration made earlier on the Castor main stent graft. Following the gradual expansion of the in-vitro fenestration by balloons of varying sizes, a covered short self-expanding stent graft of the appropriate size was deployed, and the patency of the self-expanding stent graft was evaluated using DSA. LCA was reconstructed, and complete cerebral perfusion was restored at this point. If the expansion of the covered short self-expanding stent graft was insufficient, the expansion balloon could be used for post-expansion to enhance its attachment to the LCA. The LSA was then about to be reconstructed. An expansion balloon was fed into the orifice of the LSA to expand the in-vitro fenestration along the guide wire in the sheath tube (Fustar™ Steerable Introducer System®, Lifetech Scientific™, Shenzhen, China) in the LSA track, being an elongated sheath coupled with a flexible guiding catheter. After introducing the Fustar sheath through the LSA, the tip was deflected to face the in-vitro fenestration made earlier on the Castor main stent graft, and the guide wire easily penetrated through the in-vitro fenestration to establish the LSA track. To complete the reconstruction, a small self-expanding coated stent of the appropriate size was implanted to reconstruct LSA. DSA was also used to confirm the patency and endoleak of the self-expanding coated stent. If the coated stent's expansion was insufficient, the balloon could be used post-expansion to increase its attachment to the LSA.
2. *Partial reconstruction*. The method is similar to total reconstruction, except that the landing zone is in zone Z1 (Fig. 4). According to the distance between SABs and the forward length of Castor (see "L3" in Fig. 2A), in-vitro fenestration can be divided into two situations: (1) in-vitro fenestration behind a single-branch of “Castor” (Fig. 4I) and (2) in-vitro fenestration in front of a single-branch of “Castor” (Fig. 4II). This study focused on a more commonly used condition, i.e., in-vitro fenestration behind a single-branch “Castor” (Fig. 4I). The sheath was inserted by retrograde puncture of the left common carotid artery. After the landing zone was clearly defined and marked by angiography, a good guidewire track was established along the aorta and LCA. After sending the main body of “Castor” along the aorta track to the marked landing zone, it was released in a flash and completely encompassed the aortic arch lesion from the landing zone to the distal descending aorta. Meanwhile, the single branch of “Castor” was instantly pulled into the LCA and released along the LCA track to restore the LCA perfusion. During this process, there was almost no cerebral ischemia-perfusion time. After the rapid reconstruction of LCA, the LSA was reconstructed in the same manner as during the total reconstruction process. Other videos show this in more detail (Additional files 1, 2, 3, 4, 5 and 6: Videos A, B, C, D, E and F).

For another situation (2), in-vitro fenestration of LCA in front of a single “Castor” branch (Fig. 4II), The main differences from situation (1) were: guidewire track was established along the aorta and LSA (not LCA). After sending the main body of “Castor” along the aorta track to the marked landing zone, the single branch of “Castor” was pulled into the LSA (not LCA). Then the LCA was reconstructed via in-vitro fenestration in the same way it was reconstructed in the total reconstruction process.

During this time, if in-vitro fenestration failed in the LCA, we would switch to in-situ fenestration or hybrid arch repair by right common carotid artery-left common carotid artery artificial vascular bypass. If in-vitro fenestration failed in the LSA, we would occlude the LSA to leakage from the in-vitro fenestration hole. If the aneurysm cavity is large and the endoleak is severe, the candy-plug technique or coil embolization could be used for false lumen occlusion [12]. Angiography was conducted upon completion of SABs reconstruction to confirm that no intra-operative complications existed. We made the protocols of at least 6-month postoperative anticoagulation using clopidogrel (75 mg qd) to prevent occluding SABs stent.

### Clinical parameters definition

Early mortality refers to postoperative deaths within 30 days. Cerebral infarction or intracerebral hemorrhage was used to diagnose stroke. Kidney Disease Improving Global Outcomes (KDIGO) guidelines were utilized to identify acute kidney injury [13]. Endoleaks were indicated as ongoing blood flow outward from graft/within aneurysm sac [14].

### Postoperative follow-up

Postoperative monitoring CTA imaging was routinely recorded one week, three months, six months, and annually following endovascular repair to assess endoleak, the patency for SABs, and migration, together with remodeling morphologies for dissection/aneurysm.

### Statistical analyses

SPSS Version 26.00(SPSS Inc., Chicago, US) was used to analyze the obtained data statistically. Data distribution was determined by Shapiro–Wilk test. Median and interquartile ranges were used to describe non-normally distributed continuous variables, whereas the mean ± standard deviation (SD) was used to describe normally distributed continuous variables.

## Results

### Baseline data

Herein, 57 eligible patients underwent this treatment between June 2018 and December 2021. Each patient receiving TEVAR was chosen with great care and experienced the revascularization of supra-aortic branches (SABs).

This study involved 20 female and 37 male participants, while the study population's average age was 67. 36 ± 11.09 years. Mean BMIs in cases were 25.60 ± 4.32 kg/m^2^. Among these patients, 43 (75.44%) had hypertension, and 13 (22.81%) had diabetes mellitus. Overall, 22 had a history of tobacco abuse, 7 had peripheral vascular disease, 7 had renal insufficiency, 1 had ischemic stroke and no one suffered from paraplegia prior to surgery.

For the types of lesions, 19 had Stanford-type non-A non-B dissection, 9 had a penetrating aortic ulcer, 20 had symptomatic thoracic aortic aneurysms, and 9 had intramural hematomas. Table 1 displays all demographic/clinical datasets.

**Table 1:** Baseline characteristics of 57 patients

Item	Data
*Demographics*	
Age (years)	67. 36 ± 11.09
Male/female	37/20
Body mass index (kg/m^2^)	25.60 ± 4.32
Tobacco abuse, n (%)	29 (50.88%)
Drinking, n (%)	15 (26.32%)
*Comorbidities, n (%)*	
Hypertension	43 (75.44%)
Diabetes	13 (22.81%)
Hyperlipidemia	8 (14.04%)
Peripheral vascular disease	7 (12.28%)
*Type of pathologies (n, %)*	
Type non-A non-B aortic dissection	19 (33.33%)
Acute dissection	4 (7.01%)
Chronic dissection	15 (26.32%)
Aortic arch aneurysm	20 (35.09%)
Intramural hematoma (IH)	9 (15.79%)
Acute IH	2 (3.51%)
Chronic IH	7 (12.28%)
Aortic ulcer	9 (15.79%)
*Type of presenting disease (n, %)*	
Chest-back pain	19 (33.33%)
Hoarseness	4 (7.02%)
Paraplegia	0 (0.00%)
Ischemic stroke	1 (1.75%)
Cough	9 (15.79%)
Dizzy	9 (15.79%)
Asymptomatic	15 (26.32%)
The proximal extension of the treated lesion, mm	36 [27,48]

### Surgical detail

Total SABs reconstruction was performed in 30 cases (including a hybrid arch repair case due to a huge aortic arch aneurysm), the IA and the LCA reconstruction was performed in 5 cases, and the LCA and the LSA reconstruction was performed in 22 patients. Among them (excluding the hybrid arch repair case due to a huge aortic arch aneurysm), the in-vitro fenestration technique for the LCA was successfully conducted in 32/34 cases (2 switched to in-situ fenestration due to the complete misalignment of the in-vitro fenestration position and the LCA). LSA was successfully conducted in 51/56 cases (3 switched to in-situ fenestration due to the complete misalignment of the in-vitro fenestration position and the LSA; 2 converted to spring coil caulking due to tortuous and angulated vessels). LCA and LSA were simultaneously successfully performed in 27/34 cases. One case of LSA filling spring coil was found to have slight endoleak by postoperative DSA. Table 2 indicates all surgical data.

**Table 2:** The surgical data of the 57 patients

Item	Data
*Type of reconstruction (n, %)*	
IA + LCA + LSA reconstruction	30/57 (52.63%)
IA + LCA reconstruction	5/57 (8.77%)
LCA + LSA reconstruction	22/57 (38.60%)
*In vitro fenestration success (n, %)	
LCA + LSA success	29/34 (85.29%)
LCA success	32/34 (94.12%)
LSA success	51/56 (91.07%)
*Operation time (mins)*	
IA + LCA + LSA reconstruction	112.20 ± 28.07
IA + LCA reconstruction	110.08 ± 26.38
LCA + LSA reconstruction	95.34 ± 20.55
Proximal landing zone diameter, mm	36.0 (30.0,38.0)
Distal landing zone diameter, mm	28.0 (24.0,30.0)
*Stents brand and size*	
Castor branches diameter, mm	12.70 ± 1.34
Castor branches length, mm	31.80 ± 2.05
*Bridging stents brand*	
Fluency plus (BD, USA)	20/53^#^ (37.74%)
diameter, mm	9.7 ± 1.9
length, mm	44.9 ± 6.3
Viabahn (W.L. Gore & Associates, USA)	33/53^#^ (62.26%)
Diameter, mm	9.9 ± 1.6
Length, mm	43.9 ± 5.8
Simultaneous abdominal endovascular treatment (n, %)	3/57 (5.26%)
Endoleak (n, %)	1/57 (1.75%)
Transfusion of blood cells (n, %)	3/57 (5.26%)
Hospital stay (days)	9.96 ± 4.02

*IA* Innominate artery; *LCA* Left carotid artery; *LSA* Left subclavian artery

*Excluding a hybrid arch repair case due to huge aortic arch aneurysm

^#^Excluding 1 hybrid arch repair case, 2 spring coil caulking cases and 1 case of abandoning bridging stent implantation

### Early outcome

No patient died during the procedure. The patients required 12. 85 ± 2.96 h of postoperative mechanical breathing on average. The patient spent 38.88 ± 10.27 h in the intensive care unit. The average hospitalization was 9.46 days, ranging from 7 to 29 days. 3 (5.26%) in-hospital deaths occurred. One patient died from an ischemic stroke on the fourth day following surgery. Another death occurred on day 17 postoperatively from gastrointestinal bleeding. The last patient passed away on day 29 post-operation from a hemodialysis-related brain hemorrhage. No neurological complications connected to fenestration happened. One patient (1.75%) was found to have had an ischemic stroke and lifelong neurological impairments due to ischemic stroke existing before the operation and died on the fourth postoperative day, as mentioned earlier. Out of two patients (3.50%) requiring hemodialysis for renal failure after surgery, one died on a postoperative day 29 from a brain hemorrhage related to the hemodialysis. Ischemic symptoms of the left arm occurred in one patient. There was no postoperative malperfusion or paralysis syndrome. Table 3 displays all postoperative in-hospital complications data.

**Table 3:** In-hospital complications of the 57 patients

Item	Data
Paraplegia (n, %)	0/57 (0.00%)
Ischemic stroke (n, %)	1/57 (1.75%)
Cerebral hemorrhage (n, %)	1/57 (1.75%)
Access vessel complication (n, %)	0/57 (0.00%)
Puncture site infection (n, %)	0/57 (0.00%)
Ischemic symptoms of the left arm (n, %)	1/57 (1.75%)
Myocardial infarction (n, %)	0/57 (0.00%)
Pulmonary infection (n, %)	4/57 (7.02%)
Renal failure (n, %)	2/57 (3.51%)
Mechanical ventilation time (hours)	12. 85 ± 2. 96
Intensive care unit stay (hours)	38.88 ± 10. 27
In-hospital aortic-related mortality (n, %)	0/57 (0.00%)
In-hospital mortality (n, %)	3/57 (5.26%)

*IA* Innominate artery; *LCA* Left carotid artery; *LSA* Left subclavian artery

### Follow up

The average duration of the follow-up was 3.75 months. On day 25 post-discharge, one patient died from acute liver failure. There was no stent migration or endoleak found. Endoleak disappeared in the original minor endoleak case of LSA filling with spring coil, as mentioned earlier. As mentioned earlier, the ischemic symptoms of the left arm also disappeared completely. Stent migration and endoleaks were not found. The aorta had positive remodeling due to false lumen thrombosis in aortic dissection cases and aneurysm lumen thrombosis in aneurysm cases, and postoperative follow-up CTA imaging verified the patency of all the reconstructed SABs. Table 4 displays all relevant follow-up information.

**Table 4:** Follow-up information of the 54 survival patients

Item	Data
Aortic rupture (n, %)	0/54
Paraplegia (n, %)	0/54
Stroke (n, %)	0/54
Ischemic symptoms of the left arm (n, %)	0/54
P-SINE (n, %)	0/54
D-SINE (n, %)	0/54
Endoleak (n, %)	0/54
Migration of stents (n, %)	0/54
False lumen thrombosis (n, %)	19/19
Positive remodeling of the aorta (n, %)	54/54
Patency of reconstruction supra-aortic branches (n, %)	54/54
Conversion to open surgery (n, %)	0/54
Secondary endovascular intervention (n, %)	0/54
Follow-up aortic-related mortality (n, %)	0/54
Follow-up mortality (n, %)	1/54*

*P-SINE* Proximal stent graft-induced new entry; *D-SINE* Distal stent graft-induced new entry

*Died of severe liver failure

## Discussion

Initially, TEVAR was approved by the US Food and Drug Administration for implantation in humans but exclusively for descending thoracic aneurysms within Ishimaru zones 3–5 [15]. The first requirement for TEVAR is a sufficient proximal landing zone from 1.5 to 2.0 cm at each end of the aortic lesion [16]. Clinically, in some patients, the tear location is adjacent to the supra-aortic branches (SABs) and may even expand into the ascending aorta. TEVAR is hard in these individuals due to the lack of a sufficient landing zone resulting from the lesions' proximity to SABs. However, indications within active employment for TEVAR across multiple lesion types increased due to the continued advancement of TEVAR technology. Even though those who underwent endovascular therapy were older, more severely ill, having additional comorbidities than those who underwent open aortic arch repair, the death rate for patients with endovascular treatment was markedly lower [17]. Thus, more and more TEVAR procedures are aimed at completing this alluring option to arch reconstruction for aortic arch aneurysm and aortic dissection [7].

Notwithstanding positive initial results for endovascular aortic arch rebuilding, only selected institutions can undertake this sort of endovascular therapy. This is because cerebral blood flow during surgery is difficult to maintain and because of the variant anatomical features of aorta pathologies.

Our center previously documented 62 cases of endovascular therapy using a conventional unbranched stent in conjunction with in-situ fenestration to entirely or partially restore the SABs, which achieved encouraging postoperative and short-term follow-up outcomes [18]. However, during the procedure, even with the advancement of brain protection and in-situ fenestration techniques, the brain blood supply only depends on a gutter space between the main stent and the periphery of the sheath inserted within ascending aorta via right IA. Cerebral perfusion is only partially restored until the in-situ fenestration of LCA is completed. During the in-situ fenestration period, cerebral ischemia is still possible. When the in-situ fenestration gets tough, the cerebral ischemia time window is lengthened, and the brain's blood supply appears to be struggling, the probability of cerebral infarction in this high-risk population increases. To summarize the above experience, we employed a unique unibody single-branched stent graft termed “Castor” in conjunction with in-vitro fenestration to overcome and perfect endovascular repair for the aortic arch and SABs. This research reviewed the clinical management of patients at our institution who underwent “Castor” combined with in-vitro fenestration to evaluate the death rate and cerebral disorder occurrences and the pros/cons of such a procedure.

Even though a potentially dangerous side effect of endovascular aortic arch repair is cerebrovascular happenings, a comparative analysis indicated endovascular surgical procedure reflected feasible-alternative for conventional open surgery concerning surgical mortality and neurological events [7], with an initial frequency of cerebrovascular accidents in endovascular aortic arch repair ranging solely across 0–5.4% [19–21]. Our study's use of “Castor” in conjunction with an in-vitro fenestration treatment and subsequent clinical outcomes brought about similar contentment and encouraging findings regarding cerebral complications and mortality.

Despite the positive results of the 62 cases treated endovascularly with conventional unbranched stents and in-situ fenestration in re-developing SABs that were previously reported [18], we may still encounter some snags so that we will give up this kind of technology in clinical practice, such as the aortic arch and SABs anatomical variations, bovine/gothic/giant arch aneurysms, and inappropriate vascular access [22]. Based upon 62 in-situ fenestration cases that had previously occurred during the total endovascular aortic arch reconstruction, we observed that the following aspects were the main sources of procedural difficulty [18].

First, in-situ fenestration of a "type III arch" is challenging. IA begins below the horizontal plane within the aortic arch inner curve, referred to as a "type III arch" [23]. The puncture sheath tip's stability against the main body stent membrane is essential for a successful in-situ fenestration. When it comes to a "type III arch, " the angle between the needle and main stent and the angle between aortic arch branches and aorta are extremely narrow. Once the needlepoint approaches the main body stent membrane, the sheath tip is rapidly dislodged. Castor, a novel unibody single-branched stent graft, was utilized to optimize the procedure. As mentioned earlier, when the main stent of “Castor” was fed into the landing zone, the single branch of “Castor” was pulled into the IA (total reconstruction) or LCA (partial reconstruction). It was then released in an instant just after the main stent of “Castor” was released to restore the IA or LCA perfusion immediately, directly omitting the step of in-situ fenestration and avoiding the embarrassing experience of "the sheath tip will easily shift or slip off" in in-situ fenestration. When performing in-vitro fenestration, a hydrophilic Stiff guidewire could easily enter through the aperture of the in-vitro fenestration we prepared earlier on the Castor main stent graft and brought in the covered short self-expanding stent graft. This avoided the challenge of "the sheath tip will easily shift or slip off" in in-situ fenestration in a "type III arch" or "steep arch."

**Fig. 5:**
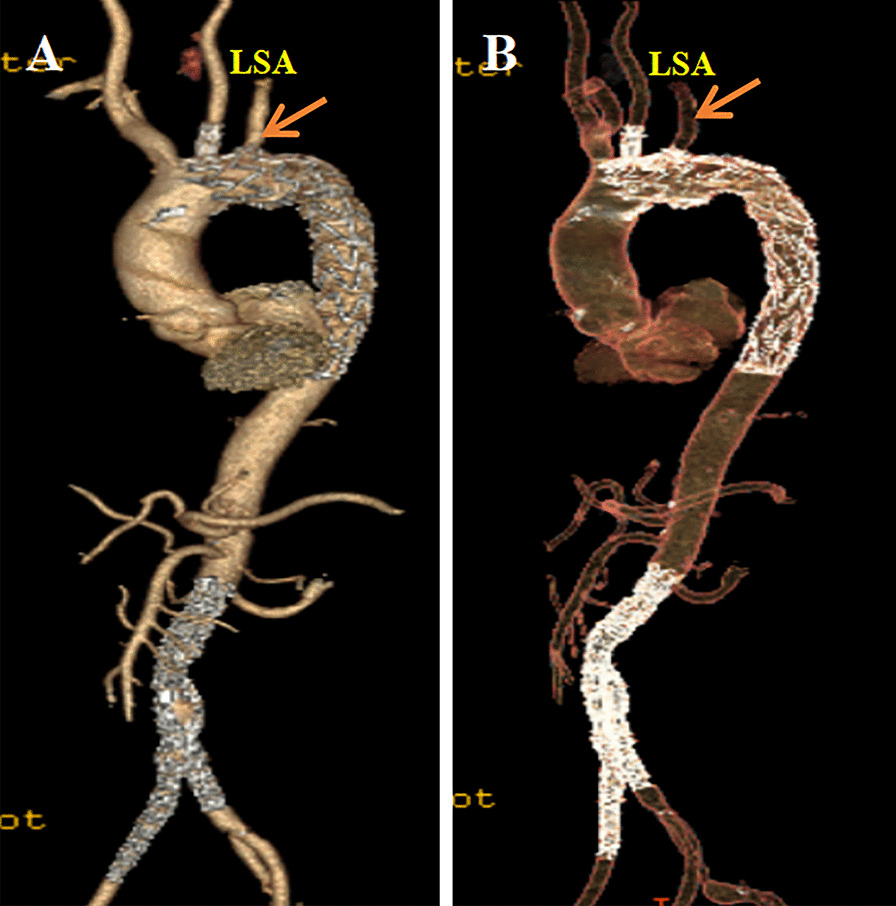
**A** A case we gave up implanting covered short self-expanding stent graft to LSA due to unsatisfactory dilation effect of in-vitro fenestration. **B** Postoperative CTA comfortingly show the perfect patency of LSA through the aperture of the in-vitro fenestration and there was no membrane leakage

In our practice, some cases failed to implant covered short self-expanding stent grafts due to difficult balloon dilation. We hypothesized that this was because the position of the aperture of the in-vitro fenestration we prepared earlier on the Castor main stent graft and the position of the arch branch vessels to be reconstructed did not overlap or did not overlap completely. The Castor stent's fabric covering was less flexible than the polytetrafluoroethylene (e-PTFE), the material typically used for in-situ fenestration. Therefore, we performed a cautious balloon dilation instead; when the dilation effect remained poor even after the pressure pump reached the pressure limit required for balloon dilation, we abandoned covered brief self-expanding stent graft implantation (Fig. 5A). However, this case was discovered to have satisfactory blood flow into the LSA through the aperture of the in-vitro fenestration we prepared upon Castor main stent graft when it was reexamined three months later, and there was no membrane leakage even though the implantation of the covered short self-expanding stent graft had failed (Fig. 5B). This is also an advantage over in-situ fenestration.

Second, in-situ fenestration is highly challenging within huge aneurysms implicating the aortic arch. Huge aortic arch aneurysm cavities render it challenging to stabilize the sheath over the main stent's fabric-based segment due to excessive gaps across SABs and the main stent, with the sheath being essential 'in-flotation' within the aneurysm cavity. The success rate for in-situ fenestration for such an arch structure is poor [20]. The risk of cerebral ischemia increases by taking a long time to reconstruct cerebral vascular branches. Herein, we used “Castor” to overcome this. As mentioned earlier, benefit from the establishment of guide wire and conduit track, the single-branch of “Castor” could be pulled into the IA (total reconstruction) or LCA (partial reconstruction) with ease as the main stent of “Castor” fed into the landing zone, even if "the aneurysm cavity involving the arch is huge." Benefiting from the aperture of the in-vitro fenestration we prepared earlier on the Castor main stent graft, a hydrophilic Stiff guidewire can be more likely to enter the in-vitro fenestration and introduce a covered short self-expanding stent graft of slightly longer length to increase the stability of the stent in this case. It is not necessary to puncture the main stent of “Castor” with a puncture needle like an in-situ fenestration, and it does not matter so much even if "the aneurysm cavity involving the arch is huge." Of course, there is also a special case: a patient with a large aneurysm complicated with severe calcification of the tumor wall. Considering the difficulty of balloon dilation and self-expanding stent graft implantation and the high risk of cerebral infarction due to the fall-off of calcification plaque caused by the process, we gave up the in-vitro fenestration and self-expanding stent implantation instead of only implanting the main stent of “Castor” with its single-branch implantation to reconstruct the IA. Then a hybrid arch repair technology by supra-aortic debranching (IA-LCA-LSA) combined with “Castor” Stent-Graft was used to restore the blood supply of LCA and LSA, which likewise demonstrated positive outcomes [24].

Third, of the three aortic arch branches, LSA anatomical form is the most challenging to in-situ fenestrate [18]. Reconstruction of LSA was proposed in our cases using in-vitro fenestration in conjunction with a covered short self-expanding stent-graft implantation for both total and partial SAB reconstruction. Although it would take longer than described above, it is possible to pull a single Castor branch into the LSA via the track (except for the situation (2) as mentioned earlier: in-vitro fenestration in front of a single branch of “Castor”). However, compared to in-situ fenestration, the likelihood of LSA reconstruction is also higher, benefiting from the aperture of the in-vitro fenestration. When performing in-situ fenestration, the LSA's anatomical variations, such as its abnormally twisted morphology, its small angle with the arch, and its narrow mounting, or accompanied by atherosclerotic plaques, rendering it challenging for sheath-tip via the brachial artery, to contact upon fabric part of the stent graft steadily [18]. However, within our investigation of in-vitro fenestration, there is no need "for the tip of the sheath via the brachial artery to steadily contact on the fabric part of the stent graft when performing in-situ fenestration." If the success of in-vitro fenestration is far beyond range, we can determine to conduct embolization of the LSA after assessing the vertebral artery advantage.

To sum up, although endovascular aortic arch repair presents a procedural challenge, we present the possibility of endovascular total aortic arch reconstruction using a novel unibody single-branched stent graft called “Castor” in combination with in-vitro fenestration technology to optimize the reconstruction process and to reduce the risk of cerebral ischemia. The present study reported almost negligible incidence in major endoleaks following endovascular aortic arch repair, and no stent obstacle was seen in the short term. The follow-up period is insufficient to rule out the possibility of late occlusion of the bridging stents, particularly in in-vitro fenestrations. This study also lacks comparison between in-vitro fenestrations and in-situ fenestrations.

### Limitations

Even with these promising outcomes, there are still certain limitations. First, only a small number of patients were examined. Second, current Castor stent models may not be adequate for all aortic arch lesions, especially for the huge IA. Third, in-vitro fenestration may cause tearing and damage to the stent graft's fabric covering [25].

## Conclusion

Endovascular aortic arch reconstruction utilizing the “Castor” unibody single-branched stent graft combined with the in-vitro fenestration is a safe, practical, effective, and reproducible therapeutic strategy for reconstructing SABs. However, the follow-up duration should be lengthened to evaluate this technique's long-term efficacy.

## Supplementary Information

**Additional file 1: Video A.** Send the main body of "Castor" to the marked landing zone and meanwhile pull the single-branch of "Castor" into the LCA.

**Additional file 2: Video B.** Release the main body of "Castor".

**Additional file 3: Video C.** Release the single-branch of "Castor" in the LCA.

**Additional file 4: Video D.** Expansion the in vitro fenestration in the face of the LSA.

**Additional file 5: Video E.** Post-expansion the implanted self-expanding coated stent.

**Additional file 6: Video F.** DSA verify the patency of the self-expanding coated stent.

## Data Availability

The data that support the findings of this study are available from Fujian Cardiac Medical Center but restrictions apply to the availability of these data, which were used under license for the current study, and so are not publicly available. Data are however available from Liang-Wan Chen author upon reasonable request and with permission of Fujian Cardiac Medical Center.

## Abbreviations

SABs: Supra-aortic branches
IA: Innominate artery
LCA: Left carotid artery
LSA: Left subclavian artery
TEVAR: Thoracic endovascular aortic repair
DSA: Digital subtraction angiography
CTA: Computed tomographic angiography
